# Automatic Gender and Age Classification from Offline Handwriting with Bilinear ResNet

**DOI:** 10.3390/s22249650

**Published:** 2022-12-09

**Authors:** Irina Rabaev, Izadeen Alkoran, Odai Wattad, Marina Litvak

**Affiliations:** Software Engineering Department, Shamoon College of Engineering, 56 Bialik St., Be’er Sheva 8410802, Israel

**Keywords:** automatic handwriting analysis, gender classification, age classification, writer’s demographics classification, bilinear CNN, bilinear ResNet

## Abstract

This work focuses on automatic gender and age prediction tasks from handwritten documents. This problem is of interest in a variety of fields, such as historical document analysis and forensic investigations. The challenge for automatic gender and age classification can be demonstrated by the relatively low performances of the existing methods. In addition, despite the success of CNN for gender classification, deep neural networks were never applied for age classification. The published works in this area mostly concentrate on English and Arabic languages. In addition to Arabic and English, this work also considers Hebrew, which was much less studied. Following the success of bilinear Convolutional Neural Network (B-CNN) for fine-grained classification, we propose a novel implementation of a B-CNN with ResNet blocks. To our knowledge, this is the first time the bilinear CNN is applied for writer demographics classification. In particular, this is the first attempt to apply a deep neural network for the *age* classification. We perform experiments on documents from three benchmark datasets written in three different languages and provide a thorough comparison with the results reported in the literature. B-ResNet was top-ranked in all tasks. In particular, B-ResNet outperformed other models on KHATT and QUWI datasets on gender classification.

## 1. Introduction

It was previously shown that handwriting varies according to several factors, including demographic and geographical region [1]. Handwriting gender and age classifications are of great interest in various fields, including psychology, historical document analysis, and handwriting biometrics. Psychological investigations have confirmed that gender [2,3] and age [4,5] can be classified based on a number of features in handwriting. Typically, male handwriting is more angular, disorderly, and slanted than female handwriting, which is more regular, ordered, and round. The authors of [5] showed a significant effect of age on writing performance. The research of Marzinotto et al. [6] reported the correlation between age and handwriting styles (length of strokes, pressure, stability between words, number of pen-ups, etc.).

This paper focuses on two tasks—automatic gender and age classification from handwriting. Although there exist various automatic approaches for gender and age prediction from handwriting, the challenges of these tasks are illustrated by the comparatively low performances of the proposed models. In addition, despite the success of CNN in classifying gender, deep neural networks were never used for the purpose of classifying *age* from handwriting. Additionally, the majority of the publications that have been published in this field concentrate on the English and Arabic languages. In this work, in addition to Arabic and English, we perform experiments on documents written in Hebrew, which has received a lot less attention.

Based on the success of the bilinear convolutional neural network (B-CNN) for fine-grained classification, we offer a novel implementation of a B-CNN [7] using ResNet instead of VGG blocks. In our previous study [8], we compared multiple deep CNNs on the gender classification task, where VGG demonstrated poor performance. Therefore, in this work, we decided to replace VGG blocks with ResNet, which showed better performance and was relatively easy to integrate within B-CNN. To the best of our knowledge, this is the first time that the B-CNN has been utilized for the classification of writer demographics. In particular, though deep learning methods were previously successfully applied for gender classification from handwriting, this is the very first time that a deep neural network is employed for age classification.

The main contributions of our work are summarized as follows: (1) We modify and apply B-CNN for document image classification; (2) This is the first attempt to apply deep learning models for *age* classification from handwritten document images; (3) We substitute VGG blocks in the B-CNN architecture by ResNet due to the VGG poor performance in our previous study on gender prediction from handwriting [8]; (4) We perform thorough experiments on three benchmark datasets consisting of handwritten documents in three different languages: Arabic, English, and Hebrew. We show that when enough training data are available, B-ResNet outperforms other methods. Namely, on the gender classification task, B-ResNet outperformed other models on the KHATT and QUWI datasets.

The rest of the paper is organized as follows. Section 2 presents the related work and overviews the B-CNN architecture. In Section 3, we describe the proposed methodology, the datasets used for the study, and the experimental settings. Section 4 presents the comparative results of the study. Finally, Section 5 summarizes and concludes the proposed research and provides future directions.

## 2. Background and Related Work

In this section, we review the related work in the field of automatic handwriting processing. Additionally, we overview the B-CNN model and explain the motivation behind choosing it for the handwriting analysis task.

### 2.1. Gender Classification

Early works on gender classification applied unsupervised methods [9,10], using Adaptive Multi-Gradient (AMG) [9], and Multi-Gradient Directional (MGD) [10] features.

Since 2012, traditional machine learning methods in general and Support Vector Machines (SVMs) in particular [11,12,13,14,15,16,17,18,19,20,21,22,23], have become most popular. Except for SVMs, such models as Decision Trees and their ensembles (Random Forests or AdaBoost) [11,13,21,24], shallow Artificial Neural Networks [11,12,22,25], Regressions [20], Naïve Bayes [21], K-nearest neighbors [11,26], Fuzzy Rule-Based Classification [16], and Discriminant Analysis [21] were applied. We also observed that attention had been paid lately to ensemble approaches [20], where several different classifiers are combined to create a master model. The majority of the aforementioned models were applied upon textural [9,11,12,13,15,16,17,18,25] and a combination of textural and shape features [14,22,23,27,28,29,30,31]. The best accuracy rates—between 77% and 82%—were achieved by the SVM classifiers with textural features [12,16,17,27].

Deep models based on Convolutional Neural Networks started to appear in gender classification works around 2018. Deep neural networks were applied as feature extractors [21], and also end-to-end pipelines, including both feature selection and classification layers [8,32,33]. The main advantage of deep networks is their ability to learn features automatically without manual engineering. In addition, CNNs have been shown to be on par or even outperforming other classifiers on gender classification task [8,33,34]. Due to their benefits in terms of performance and usability, deep networks have recently emerged as a leader in various computer vision applications, including handwriting analysis.

### 2.2. Age Classification

In contrast to the gender classification task, not many works reported on automatic age classification, while in most of them age was only one of many demographic features identified from handwriting documents.

Bouadjenek et al. [15] applied an SVM classifier on two gradient features for a writer’s gender, handedness, and age range prediction. Three SVM predictors, each applied on a specific data feature, were subsequently combined in [16,35] to identify a writer’s gender, age range, and handedness. Emran et al. [36] investigated different classifiers—K-Nearest Neighbors, Random Forests, and SVM—using various visual appearance features for the prediction of a writer’s age, gender, and handedness.

Only a few works developed models solely for age prediction. Upadhyay and Singh [37] studied the estimation of age through handwriting characteristics in females and found that such characteristics as slant, alignment, spacing, hesitation marks, tremor, and speed are really valuable and helpful for age determination. Zouaoui et.al [38] investigated the co-training approach for age range prediction from handwriting analysis. The authors proposed several descriptors for feature generation and applied an SVM predictor for classification. Basavaraja et al. [39] proposed a new unsupervised method for age estimation using handwriting analysis with Hu invariant moments, disconnectedness features, and k-means clustering.

In [40], the efficacy of using the dynamic features generated by users of smartphones and tablets to automatically identify their age group was examined. The study with the KNN classifier provides evidence that it is possible to detect user age groups based on the words they write with their fingers on touchscreens. Research in [41] applied SVM and Random Forests to automatically classify people as adults or children based on their handwritten data, collected using a pen tablet.

The best accuracy (up to 81%) was achieved by the SVM classifier with textural features [16], leaving much room for performance improvement in age prediction from handwriting. As can be seen, all works utilized feature engineering in conjunction with conventional classifiers. Deep learning algorithms for age classification have not been used in any research.

### 2.3. Bilinear CNNs

Bilinear Convolutional Neural Networks (B-CNNs) are a straightforward and efficient architecture for fine-grained visual recognition that, according to Lin et al. [7], generalize a number of orderless texture descriptors, including the Fisher vector and VLAD. These networks capture localized feature interactions in a translationally invariant manner by representing an image as a pooled outer product of features derived from two CNNs. The outer product is produced by the bilinear pooling layer. The idea behind this layer is that the calculated feature interactions enable us to recognize more precise image details. By utilizing the higher-order information acquired in the form of pairwise correlations between features, bilinear pooling offers an efficient method to fuse representative features [42]. B-CNNs are end-to-end trainable and fall within the category of orderless texture representations.

Because textural features were widely used in handwriting analysis [9,11,12,13,15,16,17,18,25], we hypothesized that B-CNN would improve the performance of gender and age prediction from the handwritten text.

The study in [7] compared B-CNNs to both exact and approximate variants of deep texture representations and explored the accuracy and memory trade-offs they offer. As CNN blocks, authors used VGG networks. The key finding was that different outer-product representations perform exceptionally well on a variety of fine-trained, texture, and scene identification tasks. A visualization of B-CNNs in [7] revealed that these models accurately represent objects as texture and that their units are associated with localized features helpful for fine-grained recognition.

## 3. Methodology

With the advancement of storage and GPU capabilities, approaches based on deep learning have become increasingly popular. Neural networks are capable of learning very powerful and perhaps counterintuitive features that human specialists may overlook. For this reason, we chose to adopt a deep neural network developed for fine-grained classification. We begin with a description of our innovative B-ResNet implementation, followed by a detailed description of the datasets and experimental settings.

### 3.1. B-ResNet

Handwriting classification is challenging due to small inter-class and large intra-class variations. Handwriting of different persons might share similar visual characteristics, while documents written by the same person can have different appearances due to various external and behavioral factors. Therefore, the proper extraction of discriminating local features describing handwriting is crucial.

In this work, we adopt Bilinear Convolutional Neural Networks (B-CNNs) [7] for fine-grained feature extraction and further classification. Inspired by insights derived from an extensive evaluation of B-CNNs on multiple tasks and datasets in [7], we decided to experiment with B-CNN architecture and apply it to the gender and age classification tasks.

**Figure 1:**
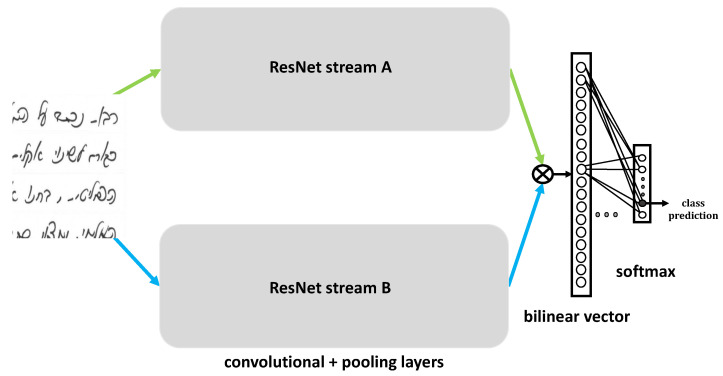
B-ResNet architecture. The input image patch contains Hebrew handwriting.

Figure 1 shows the architecture of B-CNN, where two parallel CNN blocks generate representation vectors for an input picture that are later concatenated to one bilinear vector, which is fed to the classification layers.

We first experimented with the original implementation of B-CNN that uses two VGG [43] parallel blocks. However, due to the poor results, we decided to replace VGG with another model. Based on our previous experience [8] with deep networks, where ResNet [44] outperformed VGG, we substituted VGG in two branches by ResNet. The well-known advantage of ResNet is the skip connections that address the vanishing gradient problem. Moreover, ResNet was relatively easy to integrate within B-CNN as compared to other deep-learning models. As expected, the combination of two identical ResNet networks truncated at the last fully connected layer outperformed the combination of VGGs. Therefore, we report its scores in Section 4. We denote our model by B-ResNet for the rest of the paper.

B-ResNet captures translationally invariant localized feature interactions by representing an image as the outer product of features extracted from two parallel ResNets. The bilinear pooling layer produces the outer product. We perform signed square-root normalization, followed by L2 normalization, which, according to [7], improves performance. For classification, we use the softmax function. B-ResNet is trainable end-to-end and naturally suitable for orderless texture representation. Two ResNets share all parameters; therefore, they have the same memory overhead and runtime as a single network. Two ResNet modules share no computations and are pretrained on ImageNet.

To the best of our knowledge, our work is the first attempt to apply the bilinear CNN model to a handwriting analysis with the purpose of gender and age classification. Moreover, this is the first work on age identification using deep learning models.

### 3.2. Datasets

**Figure 2:**
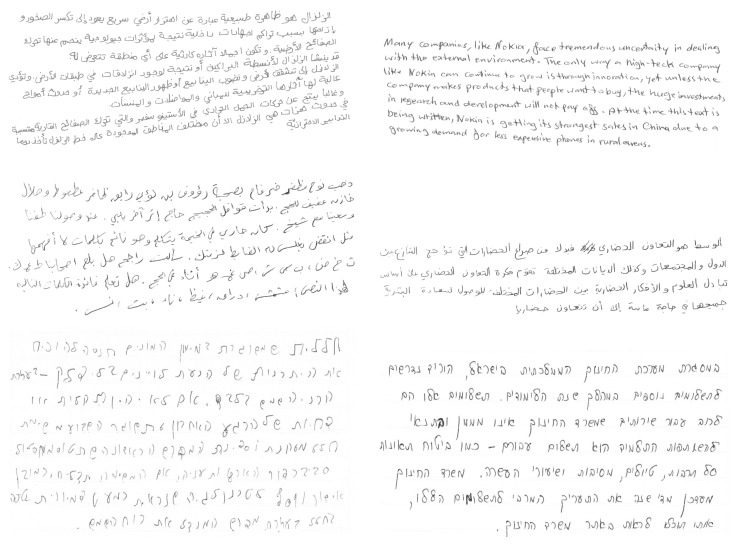
Sample images from the QUWI dataset (top) with Arabic and English handwriting, the KHATT dataset (middle) with Arabic handwriting, and the HHD dataset (bottom) with Hebrew handwriting.

For the experiments, we used KHATT, QUWI, and HHD datasets, which are publicly available. Together these datasets consist of documents written in three different languages: Arabic, English, and Hebrew. The KHATT dataset provides gender and age labels; the QUWI and the HHD datasets provide gender labels. Below we describe each one of the datasets, and Figure 2 illustrates sample images from each.

**The KHATT (KFUPM Handwritten Arabic Text)** dataset (http://khatt.ideas2serve.net/KHATTDownload.php, accessed on 1st March 2022) [45,46] consists of handwritten forms written in Arabic by 1000 writers. The ground truth contains gender (male/female) and age group (“<15”, “16–25”, “26–50”, “>50”). The forms were filled in mostly by high school and university students. Each writer contributed five paragraphs: two randomly selected paragraphs from 12 categories, two minimal text paragraphs covering all Arabic letter forms, and one free-text paragraph.

**The QUWI (Qatar University Writer Identification) dataset** was introduced in [47]. It contains handwritten documents in Arabic and English. The full dataset contains the handwriting of 1017 writers; each writer contributed four pages—two written in English and two in Arabic. The subsets of the QUWI dataset were used in several competitions—ICDAR 2013, ICDAR 2015, and ICFHR 2016 [27,28,29]. In this work, we utilized the ICDAR2013 subset of QUWI, which is publicly available on Kaggle (https://www.kaggle.com/c/icdar2013-gender-prediction-from-handwriting/data, accessed on 21 January 2021). The ICDAR 2013 subset of QUWI includes documents written by 475 writers—221 males and 254 females—and is divided into training and test sets with 282 and 193 writers respectively. There are three alternative configurations used: training and testing on English samples, training and testing on Arabic samples, and training and testing on samples written in both English and Arabic.

**The HHD (Handwritten Hebrew Dataset)** was introduced in [48]. The HHD_gender (https://doi.org/10.5281/zenodo.4729908, accessed on 30 April 2021) subset of the HHD dataset includes 702 forms, filled in Hebrew by 351 men and 351 women. Each participant volunteered demographic information and copied a paragraph of text that was printed above the text field. The form was selected at random from 50 variations and contained an average of 62 words. The HHD_gender dataset is split into training (80%), validation (10%), and test (10%) sets.

### 3.3. Experimental Settings

In our initial experiments, we utilized the bilinear CNN provided with the [7] paper (http://vis-www.cs.umass.edu/bcnn, accessed on 18 January 2022). However, the classification accuracies for both tasks were low (around 50%). We hypothesized that the VGG model used in each branch of the bilinear CNN is not the ideal option for our tasks. The original model was built and trained for general image fine-grained classification, e.g., to differentiate between different bird species or car models. In our domain, we work with document images that are very different from general images. In addition, in our previous study [8], VGG demonstrated poor performance on the gender classification task in comparison to several deep learning models. We decided to replace the VGG model in each branch of B-CNN with ResNet, which showed much better performance and was relatively easy to integrate within B-CNN.

**Figure 3:**
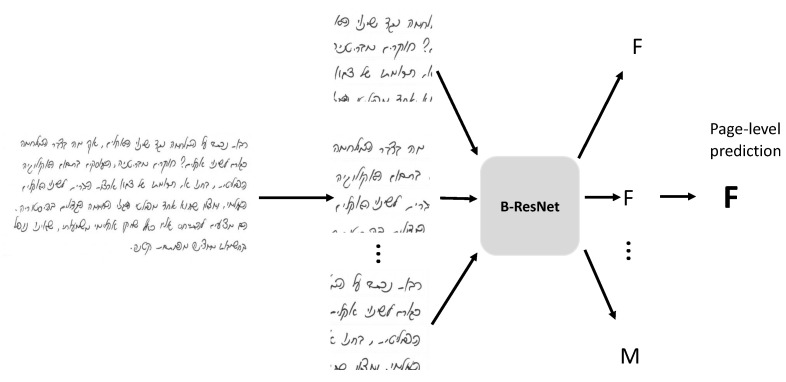
The pipeline of the classification procedure. F and M stand for female and male, respectively. The input image contains Hebrew handwriting.

The models were trained on patches extracted from document images. For accurate classification, a document image patch needs to include enough handwriting features. Based on our earlier work [8], a patch with three to four text lines should be sufficient. In our experiments, a patch size of 400×400 was sufficient. The patches were extracted by moving a sliding window at a stride of 200 pixels in vertical and horizontal directions. The average number of extracted patches was between 16 and 19. The patches were resized to the input size 448 as expected by the ResNet implementation and normalized with respect to mean and standard deviation. The weights of the two ResNet blocks were pretrained on ImageNet, while the last added layers were randomly initialized. We used a learning rate of 0.1, weight decay $1\times 10^{-8}$, and SGD optimizer. Classification results were evaluated by page-level accuracy, which is computed by taking the majority vote on the predictions of all patches from the page. Figure 3 illustrates the classification pipeline.

## 4. Results and Discussions

For gender classification, we experimented with the KHATT, QUWI (ICDAR 2013), and HHD datasets. For the age classification, we utilized the KHATT dataset. To make a consistent comparison with the results published in the literature, in all the experiments, we followed the official split into training, validation, and test sets provided with each dataset and applied the same evaluation protocol.

### 4.1. Gender Classification

In this section, we report the results for gender classification on the KHATT, QUWI, and HHD datasets. Table 1 lists the total amount of images in each of them. We added to Table 1 a ‘majority probability’ column because the datasets are imbalanced. The majority probability indicates the random guess accuracy.

#### 4.1.1. The Results on the KHATT Dataset

The KHATT dataset is provided with its official split: training (70%), validation (15%), and testing (15%) sets. For the experiment, we utilized the full dataset—2000 paragraph images written by 1000 writers. The results are presented in Table 2. We found only one additional study [33] that utilized the *full* KHATT dataset—1000 writers and 2000 paragraph images. Additional works [9,15,16,17,34,49] utilized only small subsets of KHATT with 75, 100, or 165 writes, and used segmented text line images, and their results are not directly comparable. We can see that B-ResNet achieves 76.17% classification accuracy—an increase by 2% in comparison to the results of [33] and much above the majority probability (majority probability indicates the results of the random guess).

#### 4.1.2. The Results on the ICDAR 2013 Dataset

Table 3 compares the results obtained for gender classification with the results reported in the literature. There are three classification configurations: training and testing on documents written in English, training and testing on documents written in Arabic, and training and testing on documents in both, English and Arabic.

We can see that the results of the B-ResNet are among the top-two results in all scenarios, being the winner in the two mono-script schemes and second place in the multi-script task. English and Arabic handwriting have very different patterns, and it can be expected the multi-script results will be lower than the mono-script. In all the cases, the achieved results are much above the random guess.

#### 4.1.3. The Results on the HHD Dataset

The HHD dataset consists of 702 document samples: 351 males and 351 females. We used the official split from [8]. Table 4 presents the results of the B-ResNet and compares them to the top results reported in [8]. We can see that the results of B-ResNet are on par with the results of [8], which all are far above the random guess. The size of the HHD is much smaller than that of the KHATT and QUWI, and the B-ResNet model has a much higher number of parameters (around 21M) in comparison to other models. Such a large number of parameters require a large dataset to train the model. We believe that training on a larger dataset will improve the B-ResNet results further.

### 4.2. Age Classification

In this part, we report the results of age classification on the KHATT dataset. To the best of our knowledge, KHATT is the only available public offline dataset that contains age labels. Similar to the gender classification, we used the official split provided with the dataset. Age labels are provided in the form of four classes: (1) “>15”, (2) “16–25”, (3) “26–50”, (4) “>50”. The major class is the age group between 15 and 25, which constitutes 64.4% of the entire dataset. Table 5 presents the KHATT split.

Comparison of results is difficult due to the fact that previous studies utilized different settings—a much smaller number of writers utilized in the experiments, text line segmented images as opposed to paragraph-level images, and a different number of age classes.

The experimental settings in the previous studies were as follows. In the studies of Basavaraja et al. [39] and Bouadjenek et al. [16], only two age ranges were utilized: “16–25 years” and “26–50 years”, 135 samples for each age range with the split 2/3:1/3 for training and testing sets. Bouadjenek et al. [15] collected 135 samples for each of the three classes: “under 15 years”, “16–25 years”, and “26–50 years”. For each class, 2/3 of the samples were used for the training step, while the remaining 1/3 were used for testing the system. All of the aforementioned studies employed segmented text line images.

Our experiments were performed on the *full* KHATT dataset with 1000 writers and 2000 *paragraph* images, classified into four ages group, as provided by the official split. We did not find any previous study that utilized the full KHATT dataset for age classification. The average accuracy on the KHATT dataset is 66.65%. The results for age classification are lower than the results for gender classification. Age classification seems to be a harder task as compared to gender classification, where only two classes are given in later. While inspecting the confusion matrix presented in Table 6, we can notice that the lowest results are for the age groups “<15” and “>50”. In the age group “<15”, only one image out of 42 was classified correctly, and in the group “>50” no image was assigned to the correct group. We attribute such low results to the fact that these two age groups are under-represented, especially the “>50” group—only 34 out of 2000 paragraphs are written by people above 50 years old.

Table 7 compares the results of different studies. Recall that previous studies used very different experimental settings—a much smaller number of writers, segmented line images as opposed to full paragraph images, and only two or three age classes—which does not make it possible to perform a fair quantitative comparison with the results of the proposed method. To compare the B-ResNet performance with the systems presented in [15,16,39], we calculated the accuracy for only two and three age classes. The B-ResNet archives the second-best result for two age classes scenario and the best result for three classes scenario, outperforming the result of [15] by almost 12%. It should be noted that in all the cases, our model was trained to classify four classes. We believe that training our model on two or three groups (instead of all four as we did) would improve its performance for those groups.

## 5. Conclusions and Future Work

This work focuses on automatic gender and age classification from handwriting. To the best of our knowledge, our work is the first attempt to apply B-ResNet—the B-CNN architecture, where ResNet is instantiated into both parallel CNN blocks—to a handwriting analysis with the purpose of gender and age classification. Moreover, this is the first work on age classification using a deep learning model. We demonstrate that, in most cases, the performance of the B-ResNet performance is superior to that of other models. In the future, we plan to incorporate the attention mechanism into the B-ResNet model. Experiments in [50] show that the attention mechanism significantly reduces the classification error on fine-grained datasets of general images. Additionally, we would like to explore the correlation between gender and age classification problems, e.g., whether age can be more accurately classified inside a certain gender group and vice versa.

## Figures and Tables

**Table 1:** The total amount of training, validation, and testing images in each dataset. The ‘majority probability’ column describes the imbalance in the dataset (percentage of the samples from larger class).

Dataset	Images	Male	Female	Majority Probability (%)	Train	Val	Test
KHATT	2000	1354	666	67.7	1400	300	300
QUWI	1900	884	1016	53.5	1128	-	772
HHD	702	351	351	50	560	72	70

**Table 2:** Gender prediction accuracy on the KHATT dataset.

Method	Accuracy (%)	Study
B-ResNet	**76.17**	proposed
ATP-DenseNet	74.1	Xue et al. [33]

**Table 3:** Comparison of classification accuracy (%) for gender classification on the ICDAR 2013 dataset.

Method	En/En	Ar/Ar	En+Ar/En+Ar	Study
B-ResNet	**88.33**	**85.23**	78.04	proposed
SVM	-	-	63.6	Tan et al. [23]
SVM	75.5	77.7	77.8	Akbari et al. [12]
ANN	69	71.9	**79.3**	Akbari et al. [12]
SVM	77.98	76.17	75	Gattal et al. [18]
SVM	-	-	66.3	Bi et al. [14]
SVM, LR, kNN (ensemble)	-	-	65.71	Maken and Gupta [20]
ATP-DenseNet	-	-	71.8	Xue et al. [33]
CNN	75	74	71	Rabaev et al. [8]
***Top ICDAR results*** (different systems)	79	74	76	retrieved from [12]

**Table 4:** Gender prediction accuracy on the HHD dataset.

Method	Accuracy (%)	Study
B-ResNet	84	proposed
Xception	**85**	Rabaev et al. [8]
EfficientNet	84	Rabaev et al. [8]
NashNet	84	Rabaev et al. [8]

**Table 5:** The KHATT split (number of images) for age classification problem. The major class is the age group between 15 and 25, which constitutes 64.4% of the entire dataset.

Age	Train	Test	Val
<15	172	42	38
16–25	894	196	198
26–50	310	56	60
>50	24	6	4

**Table 6:** The confusion matrix for age classification on the KHATT dataset.

	<15	16–25	26–50	>50
**<15**	1	37	4	0
**16–25**	0	168	26	0
**26–50**	0	29	29	0
**>50**	0	3	3	0

**Table 7:** Comparison of accuracy scores for age classification accuracy on the KHATT dataset for different numbers of age classes.

Method	Acc (%)	Study	Settings
**Two age classes**			
Hu moments and k-means clustering	64.44	[39]	line images, 270 writers in total
SVM & Fuzzy MIN-MAX rules combination	81.11	[16]	line images, 270 writers in total
B-ResNet	78.17	proposed	paragraph images, the full dataset
**Three age classes**			
SVM	55.55	[15]	line images, 405 writers in total
B-ResNet	67.30	proposed	paragraph images, the full dataset
**Four age classes**			
B-ResNet	66.65	proposed	paragraph images, the full dataset

## Data Availability

Not applicable.
